# The arthritis severity locus *Cia5a* regulates the expression of inflammatory mediators including Syk pathway genes and proteases in pristane-induced arthritis

**DOI:** 10.1186/1471-2164-13-710

**Published:** 2012-12-19

**Authors:** Max Brenner, Pércio S Gulko

**Affiliations:** 1 Laboratory of Experimental Rheumatology, Center for Genomics and Human Genetics, The Feinstein Institute for Medical Research, 350 Community Drive Room 1240, Manhasset, 11030, NY, USA; 2 The Elmezzi Graduate School for Molecular Medicine, Manhasset, USA

**Keywords:** Rheumatoid arthritis, Articular damage, Autoimmune

## Abstract

**Background:**

*Cia5a* is a locus on rat chromosome 10 that regulates disease severity and joint damage in two models of rheumatoid arthritis, collagen- and pristane-induced arthritis (PIA). In this study, we aimed to identify cellular and molecular processes regulated by *Cia5a* using microarray-based gene expression analysis of synovial tissues from MHC identical DA (severe erosive disease) and DA.F344(Cia5a) congenics (mild non-erosive disease) rats.

**Results:**

Synovial tissues from six DA and eight DA.F344(Cia5a) rats were analyzed 21 days after the induction of PIA using the Illumina RatRef-12 BeadChip (21,922 genes) and selected data confirmed with qPCR. There was a significantly increased expression of pro-inflammatory mediators such as *Il1b* (5-fold), *Il18* (3.9-fold), *Cxcl1* (10-fold), *Cxcl13* (7.5-fold) and *Ccl7* (7.9-fold), and proteases like *Mmp3* (23-fold), *Mmp9* (32-fold), *Mmp14* (4.4-fold) and cathepsins in synovial tissues from DA, with reciprocally reduced levels in congenics. mRNA levels of 47 members of the Spleen Tyrosine Kinase (*Syk*) pathway were significantly increased in DA synovial tissues compared with DA.F344(Cia5a), and included *Syk* (5.4-fold), Syk-activating receptors and interacting proteins, and genes regulated by *Syk* such as NFkB, and NAPDH oxidase complex genes. Nuclear receptors (NR) such as *Rxrg*, *Pparg* and *Rev-erba* were increased in the protected congenics, and so was the anti-inflammatory NR-target gene *Scd1* (54-fold increase). *Tnn* (72-fold decrease) was the gene most significantly increased in DA.

**Conclusions:**

Analyses of gene expression in synovial tissues revealed that the arthritis severity locus *Cia5a* regulates the expression of key mediators of inflammation and joint damage, as well as the expression of members of the *Syk* pathway. This expression pattern correlates with disease severity and joint damage and along with the gene accounting for *Cia5a* could become a useful biomarker to identify patients at increased risk for severe and erosive disease. The identification of the gene accounting for *Cia5a* has the potential to generate a new and important target for therapy and prognosis.

## Background

Rheumatoid arthritis (RA) is a common, chronic and potentially debilitating form of autoimmune erosive arthritis. Advances in the understanding of RA pathogenesis have led to the development of new and better treatments [1-3]. Yet, sustained remission is still rarely achieved [4], and more effective therapies are needed.

The identification of genes implicated in the regulation of arthritis severity and articular damage has the potential to generate new and potentially better targets for therapies aimed at preserving joint architecture and function, and reducing the risk of developing joint deformities. Yet, little is known about those genes [5], and the large cohorts of RA patients used in genome-wide association studies for susceptibility were not designed to address disease severity and articular damage.

We have previously identified several disease severity and articular damage quantitative trait loci (QTL) in rat models of RA [6-10]. Using a combination of positional cloning and functional studies that include transcriptome analyses of synovial cells and synovial tissues we are beginning to understand the molecular processes regulating arthritis severity and joint damage in pristane- and collagen-induced arthritis (PIA and CIA) [10-14]. Similar strategies have been successfully used to identify other autoimmunity genes in rodent models [15,16].

*Cia5a* is a 20.6Mb QTL on rat chromosome 10 that regulates arthritis severity, cartilage and bone damage, synovial hyperplasia and inflammation in both PIA and CIA [9,10]. In the present study we used synovial tissues from arthritis-protected DA.F344(Cia5a) congenics and from arthritis-susceptible and MHC-identical DA rats in a microarray analysis of gene expression. We determined that the *Cia5a* locus regulates the expression of several genes central to RA pathogenesis and joint damage, such as cytokines *Il1b* and *Il18*, chemokines, proteases, mediators of the synthesis of reactive oxygen species and prostaglandins, and genes involved in Toll-like receptor signaling. Additionally, the expression of 47 members of the *Syk* kinase pathway genes, including *NFκB* genes were significantly regulated by the *Cia5a* locus. Furthermore, the presence of F344 alleles at the *Cia5a* interval was associated with increased expression of anti-inflammatory genes, including nuclear receptors and *Timp3*, suggesting that the *Cia5a* locus contains a gene involved in maintaining an inflammation-free synovial tissue.

## Results

### DA.F344(Cia5a) congenics develop a mild form of PIA with a distinct pattern of gene expression compared with DA rats

**Figure 1:**
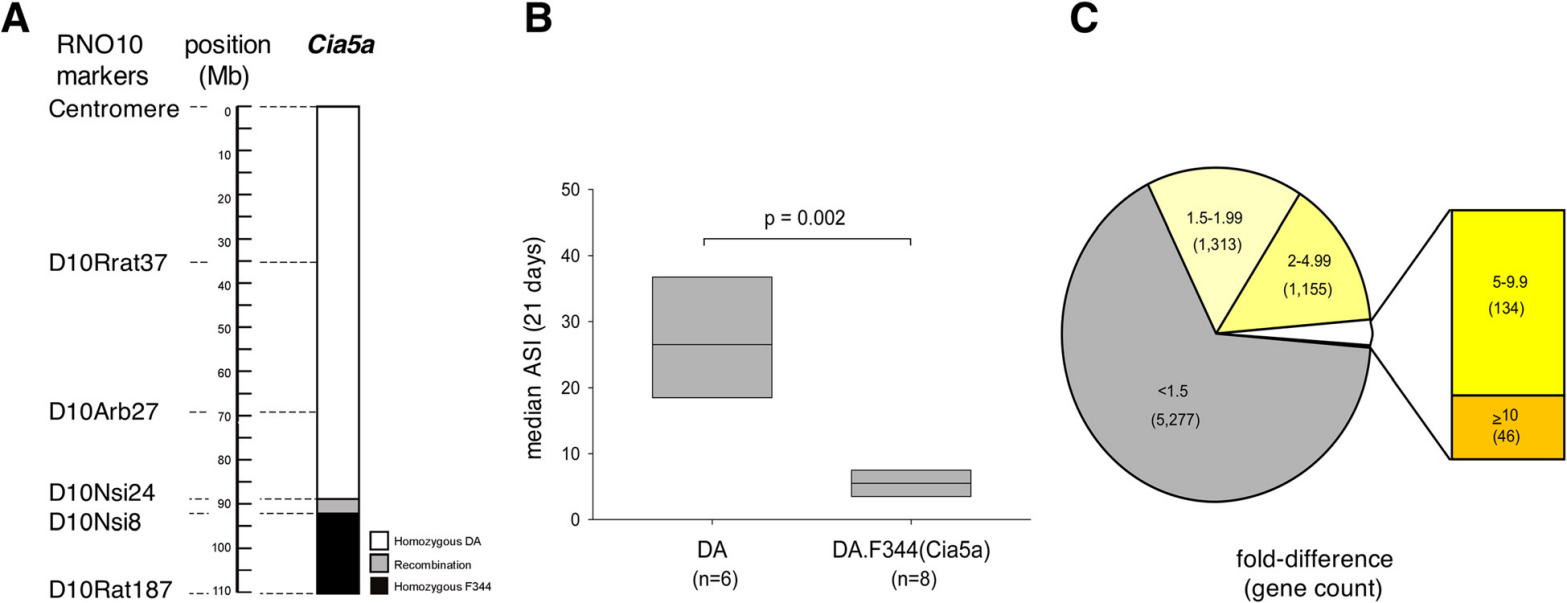
**DA and DA.F344(Cia5a) rats differ in arthritis severity and have different synovial gene expression profiles. **(**A**) Map of the *Cia5a* locus on rat chromosome 10, and the congenic interval boundaries (black=homozygous for F344 alleles; white=homozygous for DA alleles; grey=recombination interval). (**B**) DA rats had severe disease at 21 days post-induction of PIA; DA.F344(Cia5a) congenics were protected and developed a significantly milder form of arthritis (p=0.002, Mann–Whitney test; boxes show the median and 25%-75% percentiles). (**C**) 7,925 genes were expressed in all synovial tissues. 2,648 (33.4%) met the 1.5-fold difference and p-value of ≤0.01 (*t*-test) for significant difference. 134 genes differed by ≥5-fold and less <10-fold, and 46 genes differed by ≥10-fold (inset).

DA.F344(Cia5a) rats developed a significantly milder form of PIA compared with DA rats [median arthritis severity score (25–75 percentiles), DA=26.5 (17–36.9), DA.F344(Cia5a)=5.5 (3.6-7.2); p=0.002, Mann–Whitney test; Figure 1A and B].

36% (7,925) of the genes in the RatRef-12 BeadChip were consistently expressed in synovial tissues. Nearly one-third of these genes (2,648) met the filtering criteria for differential expression (fold-difference ≥1.5 and p≤0.01). The presence of F344 alleles at the *Cia5a* interval, as in DA.F344(Cia5a) congenic rats, was associated with increased expression of 1,241 genes and reduced expression of 1,407 genes compared with DA. 134 genes had a ≥5-fold difference between strains (Figure 1C). 46 genes had a ≥10-fold difference in expression, of which 19 were increased and 27 decreased in congenics, compared with DA (Tables 1 and 2).

**Table 1:** Genes with ≥10-fold reduction in expression in DA.F344(Cia5a) compared with DA

**Symbol**	**Name**	**Entrez Gene ID**	**Fold reduction**	***p*-value**
*Tnn*	Tenascin N	304913	71.69	8.6x10^-13^
*Mmp9*	Matrix metallopeptidase 9	81687	32.79	1.4x10^-9^
*Cdc2*	Cell division cycle 2, G1 to S and G2 to M	54237	24.16	1.6x10^-5^
*Mmp3*	Matrix metallopeptidase 3	171045	23.94	5.0x10^-5^
*Ccnb2*	Cyclin B2	363088	22.95	5.0x10^-6^
*Cthrc1*	Collagen triple helix repeat containing 1	282836	17.93	3.2x10^-9^
*Col12a1*	Collagen, type XII, alpha 1	25683	16.25	7.6x10^-8^
*Slpi*	Secretory leukocyte peptidase inhibitor	84386	16.16	6.4x10^-4^
*Spc24*	SPC24, NDC80 kinetochore complex component, homolog	363028	14.72	2.5x10^-6^
*Emilin1*	Elastin microfibril interfacer 1	298845	13.55	2.4x10^-6^
*Prc1*	Protein regulator of cytokinesis 1	308761	13.08	1.6x10^-5^
*Emb*	Embigin	114511	12.82	1.4x10^-5^
*Nuf2*	NUF2, NDC80 kinetochore complex component, homolog	304951	12.52	2.5x10^-6^
*Lbp*	Lipopolysaccharide binding protein	29469	11.82	8.0x10^-5^
*Cks2*	CDC28 protein kinase regulatory subunit 2	498709	11.62	5.9x10^-6^
*Wisp1*	WNT1 inducible signaling pathway protein 1	65154	11.59	9.0x10^-8^
*Cxcl1*	Chemokine (C-x-C motif) ligand 1	81503	10.88	4.3x10^-4^
*Steap1*	Six transmembrane epithelial antigen of the prostate 1	297738	10.61	1.7x10^-4^
*LOC687334*	Similar to cytoskeleton associated protein 2	687334	10.16	9.1x10^-6^

**Table 2:** Genes with ≥10-fold increased expression in DA.F344(Cia5a) compared with DA *

**Symbol**	**Name**	**Entrez Gene ID**	**Fold increase**	***p*-value**
***Scd1***	**Stearoyl-Coenzyme A desaturase 1**	246074	54.62	8.8x10^-5^
*Mpz*	Myelin protein zero	24564	39.80	7.7x10^-5^
*Akr1c19*	Aldo-keto reductase family 1, member C19	307096	26.45	3.0x10^-6^
*Nnat*	Neuronatin	94270	22.08	5.2x10^-7^
*Ces3*	Carboxylesterase 3	113902	21.06	5.4x10^-6^
*Mup5*	Major urinary protein 5	298107	18.41	9.5x10^-5^
*LOC688457*	Similar to Major urinary protein precursor (MUP)	688457	17.42	1.7x10^-4^
*Abcd2*	ATP-binding cassette, sub-family D (ALD), member 2	84356	16.95	4.2x10^-6^
*S100b*	S100 calcium binding protein B	25742	16.81	8.9x10^-8^
*Tshr*	Thyroid stimulating hormone receptor	25360	16.61	1.8x10^-7^
*LOC689147*	Hypothetical protein LOC689147	689147	15.84	7.9x10^-6^
*Thrsp*	Thyroid hormone responsive	25357	15.76	1.2x10^-5^
*LOC259244*	Alpha-2u globulin PGCL3	259244	15.74	1.8x10^-4^
*Mup4*	Major urinary protein 4	362527	15.38	9.9x10^-5^
*Omd*	Osteomodulin	83717	13.82	5.8x10^-6^
*Plekhb1*	Pleckstrin homology domain containing, family B (evectins) member 1	64471	13.26	2.9x10^-5^
*Ankrd5*	Ankyrin repeat domain 5	296184	13.14	4.3x10^-5^
***Cidea***	**Cell death-inducing DNA fragmentation factor,** **a subunit-like effector A**	291541	12.91	2.4x10^-6^
*Atp1a2*	ATPase, Na+/K+ transporting, alpha 2 polypeptide	24212	12.62	6.9x10^-5^
***Pck1***	**Phosphoenolpyruvate carboxykinase 1 (soluble)**	362282	11.95	2.4x10^-5^
*Mrap*	Melanocortin 2 receptor accessory protein	288271	11.80	1.8x10^-7^
*Timp3*	TIMP metallopeptidase inhibitor 3	25358	11.48	2.9x10^-9^
*MGC72973*	Beta-glo	361619	11.38	1.4x10^-6^
*Plp1*	Proteolipid protein 1	24943	11.33	1.3x10^-5^
***Plin***	**Perilipin**	25629	10.99	9.1x10^-7^
***Adipoq***	**Adiponectin, C1Q and collagen domain containing**	246253	10.96	3.3x10^-6^
*Pcbd1*	Pterin-4 a-carbinolamine dehydratase/dimerization cofactor of hepatocyte nuclear factor 1 a	29700	10.74	5.8x10^-8^

* **Bold**= nuclear receptor-inducible gene.

### Expression of pro-inflammatory genes, proteases (including matrix metalloproteases, MMPs) and adhesion molecules was significantly increased in DA and decreased in DA.F344(Cia5a)

**Figure 2:**
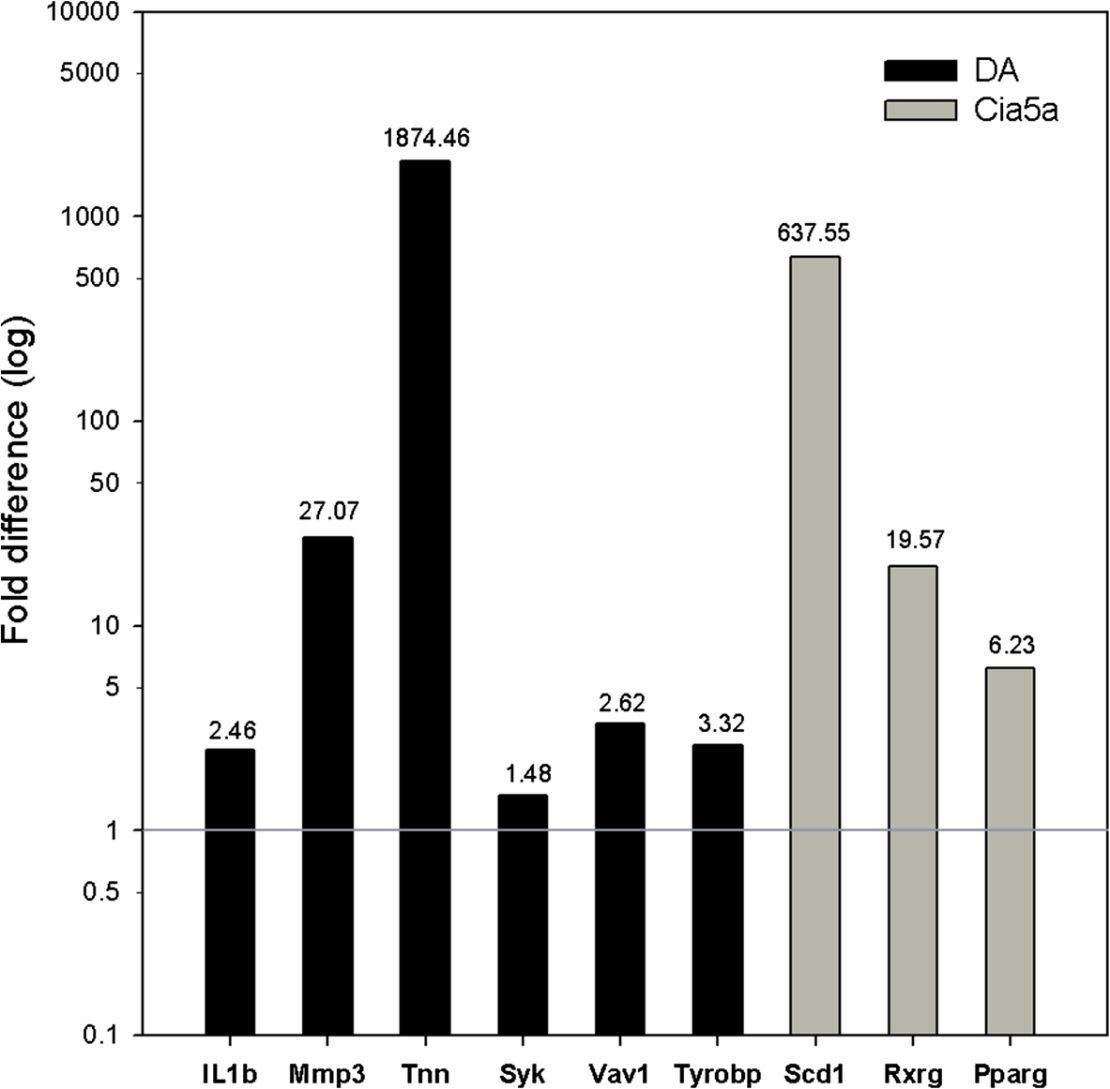
**qPCR validation of the microarray results. **Genes expressed in increased levels in DA (six genes, black bars) and genes expressed in increased levels in DA.F344(Cia5a) congenics (three genes, grey bars) were selected for qPCR confirmation. Fold-differences were log-transformed. The same RNAs used in the microarray experiments were used for qPCR. *ΔCt* was used for statistical analyses; all genes had p≤0.04, *t*-test.

The 1,407 genes with increased expression in DA and reciprocally decreased expression in DA.F344(Cia5a) congenics included pro-inflammatory cytokines and chemokines implicated in arthritis pathogenesis such as *Il1b* (5.17-fold on microarray, and 2.46-fold on qPCR), *Il18*, *Mif*, *Ccl2*, *Ccl7* and *Cxcl13* (Table 3 and Additional file 1: Table S3 and Additional file 2: Table S4). Genes with significantly decreased expression in congenics also included those implicated in the development of cartilage and bone erosions such as MMPs (*Mmp3* [24-fold], *Mmp9* and *Mmp14*), and other proteases (cathepsins D, E, K and S) (Table 3 and Figure 2). Interestingly, *Syk* (see below) has been shown to regulate the expression of differentially expressed MMPs such as *Mmp3*[17] and *Mmp9*[18], further suggesting a potential central role for *Syk* in arthritis and a Syk-regulatory effect of *Cia5a*. Components of the extracellular matrix (ECM; *Cthrc1*, *Col12a1*, *Emilin1*) also had reduced expression in congenics, and together with the levels of proteases suggested that there was reduced matrix turnover and reduced degradation, compared with arthritic DA rats (Table 3).

**Table 3:** Mediators of inflammation and articular damage up-regulated in DA synovium and down-regulation in DA.F344(Cia5a)

**Gene Symbol**	**Gene Name**	**Entrez Gene ID**	**Fold DA/Cia5a**	p-value*
***Cytokines and chemokines***				
*Il1b*	interleukin 1 beta	24494	5.17	0.002
*Il18*	interleukin 18	29197	3.91	0.0002
*Ltb*	lymphotoxin beta	361795	3.77	0.0002
*Mif*	macrophage migration inhibitory factor	81683	2.37	0.0002
*Aif1*	allograft inflammatory factor 1	29427	2.48	0.0001
*Ccl2*	chemokine (C-C motif) ligand 2	24770	3.95	0.01
*Ccl7*	chemokine (C-C motif) ligand 7	287561	7.90	0.002
*Cxcl1*	chemokine (C-X-C motif) ligand 1	81503	10.88	0.0004
*Cxcl13*	chemokine (C-X-C motif) ligand 13	498335	7.53	0.000004
***Proteases***				
*Mmp3*	matrix metallopeptidase 3	171045	23.94	0.0001
*Mmp9*	matrix metallopeptidase 9	81687	32.79	0.000000001
*Mmp14*	matrix metallopeptidase 14	81707	4.46	0.000000004
*Mmp19*	matrix metallopeptidase 19	304608	5.63	0.0000003
*Ctsc*	cathepsin C	25423	2.64	0.00007
*Ctsd*	cathepsin D	171293	1.90	0.000002
*Ctse*	cathepsin E	25424	2.03	0.0002
*Ctsk*	cathepsin K	29175	3.20	0.0000003
*Ctss*	cathepsin S	50654	1.68	0.002
***Extra-cellular matix***				
*Cthrc1*	collagen triple helix repeat containing 1	282836	17.93	0.000000003
*Col12a1*	collagen, type XII, alpha 1	25683	16.25	0.00000008
*Emilin1*	elastin microfibril interfacer 1	298845	13.55	0.000002
***Adhesion molecules***				
*Itga5*	integrin alpha 5 (fibronectin receptor alpha)	315346	3.47	0.00000001
*Itgam*	integrin alpha M	25021	3.58	0.0001
*Itgav*	integrin alpha V	296456	1.61	0.0004
*Itgb2*	integrin beta 2	309684	3.44	0.0001
*Itgb7*	integrin, beta 7	25713	3.86	0.002
*Cd44*	Cd44 molecule	25406	1.83	0.005
*Cdh11*	cadherin 11	84407	1.87	0.003
	***Toll-like receptors and regulators of their activity***			
*Cd14*	CD14	60350	2.13	0.001
*Irak4*	interleukin-1 receptor-associated kinase 4	300177	1.65	0.0004
*Lpb*	lipopolysaccharide binding protein	29469	4.36	0.0003
*Myd88*	myeloid differentiation primary response gene 88	301059	1.59	0.002
*Pycard*	PYD and CARD domain containing	282817	1.95	0.0004
*Tlr2*	toll-like receptor 2	310553	4.36	0.000
*Tlr6*	toll-like receptor 6	305353	1.76	0.001
	***Prostaglandin and leukotriene synthesis***			
*Ptgs2*	prostaglandin-endoperoxide synthase 2	29527	9.73	0.0006
*Pla2g4a*	phospholipase A2, group IVA (cytosolic, calcium-dependent)	24653	2.04	0.0004

*t-test.

Adhesion molecules required for leukocyte migration into the synovium were increased in DA synovial tissues and decreased in DA.F344(Cia5a), including integrins *Itga5*, *Itgam*, *Itgb2*, *Itgb7*, and *Cd44* (Table 3). Cadherin-11 (*Cdh11*), a FLS-specific gene required for cell-cell interactions and implicated in FLS invasion and synovial hyperplasia was also decreased in DA.F344(Cia5a) congenics, consistent with the non-hyperplastic synovial tissue previously described in this strain, as opposed to the highly hyperplastic synovial tissue seen in DA [10].

The gene with the most significantly increased expression in DA versus DA.F344(Cia5a) was *Tnn* (Tenascin N; Table 1 and Figure 2). *Tnn* has been implicated in osteogenesis and angiogenesis but not in arthritis or inflammation.

These results demonstrate that DA rats with PIA have increased synovial expression of many genes implicated in RA pathogenesis, further validating the molecular similarities between PIA and RA, and underscoring the potential relevance of both *Cia5a* in arthritis pathogenesis and the present study in discovering new key genes and pathways regulating arthritis.

### Increased expression of members of the *Syk* (spleen tyrosine kinase) pathway in DA synovial tissues, and reciprocally decreased expression in DA.F344(Cia5a)

**Figure 3:**
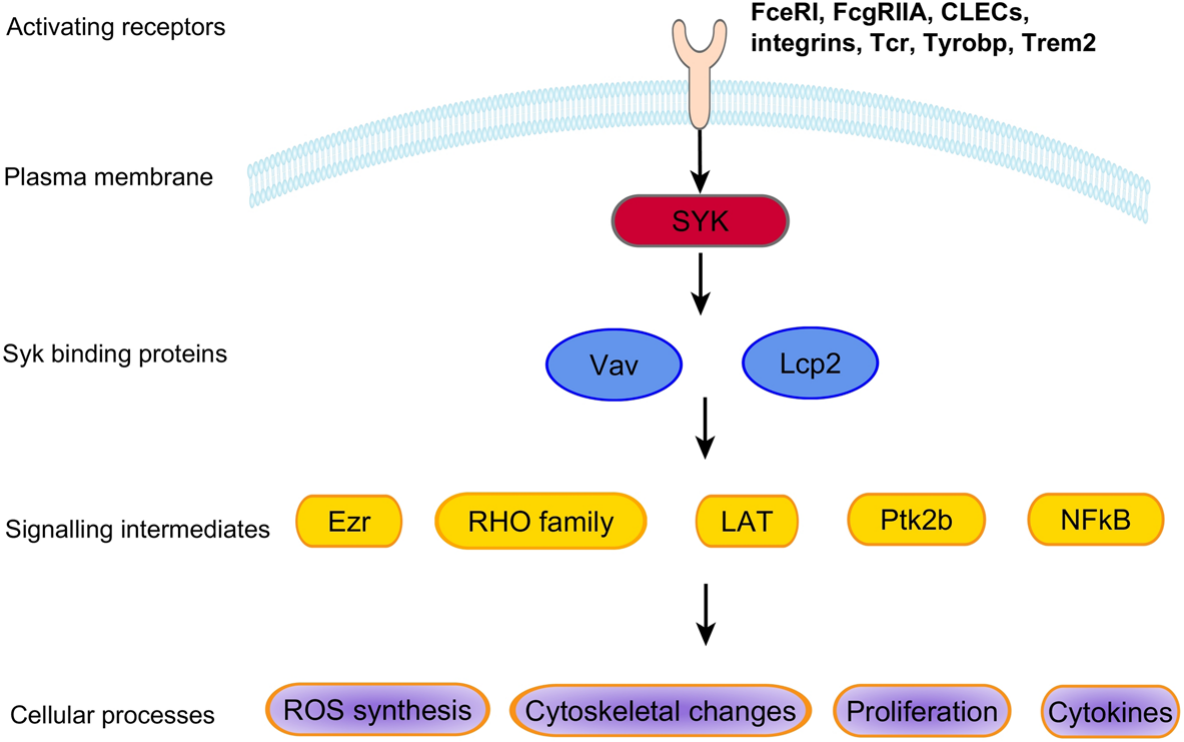
**Syk pathway genes and Syk-regulated cellular processes. **Genes, gene families and cellular processes with members expressed in increased levels in DA synovial tissues, and significantly reduced levels in DA.F344(Cia5a) congenics.

47 members of the *Syk* pathway were expressed in significantly increased levels in DA, and in reduced levels in congenics (Table 4, and Figures 2 and 3). These included: **a**) *Syk*-activating receptors such as *Fcgr2a*, *Fcer1g*, integrins (*Itga5*, *Itgb2*, *Itgam*), c-lectin receptors (*Clec4a3*, *Clec7a* [Dectin 1], *Clec11a*), *Trem2* and *Dap12* (*Tyrobp*), **b**) *Syk* (5.4-fold) itself, **c**) Syk-interacting and downstream signaling genes including *Vav1*, *Lcp2* (*Slp76*), *Ptk2b* (*Pyk2*), *Lat*, *Rac2*, and *Ezr* (*Vil2*), and **d**) genes belonging to pathways activated by *Syk* and implicated in arthritis pathogenesis and synovial hyperplasia and pannus formation such as NFκB pathway genes (*Fadd*, *Ikbkb*, *Nfkb1*, *Nfkb2*), cytokines (*Il1b*, *Ltb*, *Mif*), genes implicated in cell proliferation (*Ccnb2*, *Cdc2, Cks2, Nuf2*), cytoskeleton regulation (*Actr3*, *Arpc4*, *Coro1b*, *Ezr*/*Vil2*, *Myo9b*, *Parva, Tubb5*), and NAPDH oxidase complex genes implicated in the production of reactive oxygen species (ROS) (*Ncf1*, *Ncf2*, *Ncf4*, *Cyba*) (Table 4).

**Table 4:** Members of the Syk kinase pathway up-regulated in DA synovium compared with down-regulation in DA.F344(Cia5a)

**Gene Symbol**	**Gene Name**	**Entrez Gene ID**	**Fold DA/Cia5a**	p-value*
***Activating receptors***				
*Fcer1g*	Fc fragment of IgE, high affinity I, receptor for; gamma polypeptide	25441	2.50	0.0004
*Fcgr2a*	Fc fragment of IgG, low affinity IIa, receptor (CD32)	116591	3.08	0.005
*Tcrg*	T cell receptor gamma locus	24821	3.32	0.0001
*Trem2*	triggering receptor expressed on myeloid cells 2	301227	2.69	0.0006
*Tyrobp*	Tyro protein tyrosine kinase binding protein	361537	4.80	0.0001
***Integrins***				
*Itgam*	integrin alpha M	25021	3.58	0.0001
*Itgav*	integrin alpha V	296456	1.61	0.0004
*Itga5*	integrin alpha 5 (fibronectin receptor alpha)	315346	3.47	0.00000001
*Itgb2*	integrin beta 2	309684	3.44	0.0001
*Itgb7*	integrin, beta 7	25713	3.86	0.002
***c-type lectin receptors***				
*Clec4a1*	C-type lectin domain family 4, member a1, Dcir4	362430	2.38	0.001
*Clec4a3*	C-type lectin domain family 4, member a3, Dcir3	362431	3.00	0.0004
*Clec7a*	C-type lectin domain family 7, member a, Dectin 1	502902	8.48	0.001
*Clec11a*	C-type lectin domain family 11, member a, Scgf	29313	4.24	0.00000008
	***Syk and Syk-binding and intermediate partners***			
Ezr**	ezrin	54319	2.42	0.0001
*Lat*	linker for activation of T cells	81511	3.83	0.000001
*Lcp2*	lymphocyte cytosolic protein 2	155918	2.02	0.007
*Ptk2b*	PTK2B protein tyrosine kinase 2 beta	50646	2.54	0.0000002
*RhoG*	ras homolog gene family, member G (rho G)	308875	1.54	0.0008
*RhoH*	ras homolog gene family, member H	305341	2.25	0.001
*Syk*	spleen tyrosine kinase	25155	5.43	0.00004
*Vav1*	vav 1 guanine nucleotide exchange factor	25156	4.60	0.0001
***NFκB genes and pathway***				
*Ikbkb*	inhibitor of kappa light polypeptide gene enhancer in B-cells, kinase beta	84351	1.60	0.0006
*Ikbke*	inhibitor of kappa light polypeptide gene enhancer in B-cells, kinase epsilon	363984	1.64	0.0001
*Nfkb1*	nuclear factor of kappa light polypeptide gene enhancer in B-cells 1	81736	1.88	0.002
*Nfkb2*	nuclear factor of kappa light polypeptide gene enhancer in B-cells 2, p49/p100	309452	1.98	0.00003
	***Cytokine and chemokine transcription§***			
*Ccl2*	chemokine (C-C motif) ligand 2	24770	3.95	0.01
*Ccl7*	chemokine (C-C motif) ligand 7	287561	7.90	0.002
*Il1b*	interleukin 1 beta	24494	5.17	0.002
*Ltb*	lymphotoxin beta (TNF superfamily, member 3)	361795	3.77	0.0002
***Cell Proliferation***				
*Cdc2*	cell division cycle 2, G1 to S and G2 to M	54237	24.16	0.00002
*Ccnb2*	cyclin B2	363088	22.95	0.000005
*Cks2*	CDC28 protein kinase regulatory subunit 2	498709	11.62	0.000006
*Prc1*	protein regulator of cytokinesis 1	308761	13.08	0.00002
*Spc24*	SPC24, NDC80 kinetochore complex component, homolog (S. cerevisiae)	363028	14.72	0.000002
***Cytoskeletal changes***				
*Actr3*	ARP3 actin-related protein 3 homolog (yeast)	81732	1.88	0.0001
*Arpc4*	actin related protein 2/3 complex, subunit 4	297518	1.61	0.00005
*Capzb*	capping protein (actin filament) muscle Z-line, beta	298584	1.64	0.0003
*Coro1b*	coronin, actin-binding protein, 1B	29474	1.68	0.0001
*Myo9b*	myosin IXb	25486	1.93	0.00003
*Tiam2*	T-cell lymphoma invasion and metastasis 2	308142	2.61	0.00003
*Parva*	parvin, alpha	57341	2.19	0.00005
	***Reactive oxygen species production***			
*Cyba*	cytochrome b-245, alpha polypeptide	79129	2.72	0.00001
*Ncf1*	neutrophil cytosolic factor 1	114553	6.00	0.0001
*Ncf2*	neutrophil cytosolic factor 2	364018	4.75	0.0003
*Ncf4*	neutrophil cytosolic factor 4	500904	2.46	0.004
*Rac2¶*	ras-related C3 botulinum toxin substrate 2 (small GTP binding protein Rac2)	366957	2.32	0.0007

Syk gene is underlined. * t-test;

§=All four chemokine and cytokine listed genes are NFkB-inducible targets.

** Ezrin is also involved in cytoskeleton regulation.

¶ Rac also regulates NFkB activity, cytoskeleton and cell proliferation.

Interestingly, genes that neutralize ROS (*Cat*, *Sod1*, *Gss*) went on the opposite direction with increased expression in congenics (Table 5).

**Table 5:** Anti-inflammatory genes and nuclear receptors up-regulated in DA.F344(Cia5a) congenics

**Gene Symbol**	**Gene Name**	**Entrez Gene ID**	**Fold difference DA/Cia5a**	p-value*
	***Anti-inflammatory and regulators of immune responses***			
*Scd1*	stearoyl-Coenzyme A desaturase 1	246074	54.62	0.0001
*Timp3*	TIMP metallopeptidase inhibitor 3	25358	11.48	0.000000003
*Adipoq*	adiponectin, C1Q and collagen domain containing	246253	10.96	0.000003
*Ptpn11*	protein tyrosine phosphatase, non-receptor type 11	25622	4.18	0.00002
*Cyp2j3*	cytochrome P450, family 2, subfamily j, polypeptide 3	313375	3.38	0.002
*Ptgis*	prostaglandin I2 (prostacyclin) synthase	25527	2.63	0.0003
*Cat*	catalase	24248	1.91	0.002
*Gss*	glutathione synthetase	25458	1.56	0.001
*Sod1*	superoxide dismutase 1, soluble	24786	1.53	0.00002
	***Nuclear receptors and an interacting protein***			
*Rxrg*	retinoid X receptor gamma, Nr2b3	83574	5.39	0.0001
*Pparg*	peroxisome proliferator-activated receptor gamma, Nr1c3	25664	5.32	0.00005
*Rev-erba*	Nr1d1	252917	3.41	0.004
*Arp1*	Nr2f2	113984	2.45	0.0002
*Nrip1*	nuclear receptor interacting protein 1	304157	2.35	0.00004
*Thrb*	thyroid hormone receptor beta, Nr1a2	24831	2.07	0.002
*Thra*	thyroid hormone receptor alpha, Nr1a1	81812	1.97	0.001
*Ncor1*	nuclear receptor co-repressor 1	54299	1.89	0.003
*Rora*	RAR-related orphan receptor alpha, Nr1f1	300807	1.88	0.0001
*Lxra*	Liver X receptor alpha, Nr1h3	58852	1.52	0.0006

* *t*-test.

Additionally, *Syk* and *Vav1* expression levels correlated with the cumulative arthritis severity score (Pearson’s correlation coefficient of 0.8 and p=0.0006 for both genes). Taken together, these observations suggest that the *Cia5a* QTL contains an arthritis gene that directly or indirectly regulates the expression of *Syk* pathway genes, providing a possible mechanistic explanation for this locus’ effect on the regulation of disease severity.

### DA.F344(Cia5a) congenics have reduced synovial expression of innate immune response-activating genes, including members of the inflammasome

Expression levels of genes implicated in innate immune responses were significantly increased in DA, and decreased in DA.F344(Cia5a). In addition to the *Syk* pathway, and mediators of ROS synthesis, and regulators of cytokine transcription such as members of the NFκB pathways discussed above, DA.F344(Cia5a) congenics also had reduced expression of AP-1 genes (*Fos* and *JunB*), *Il1b* and other members of the inflammasome (*Card11*, *Nalp3* [both detected only in DA], and *Pycard*). Pattern recognition receptors such as *Zbp1* and *Lgp2* (both detected only in DA), and components of the toll-like receptor (TLR) pathway (*Cd14*, *Ikbke*, *Irak4*, *Lbp*, *MyD88*, *Tlr2*, *Tlr6*, *Ticam1*; Table 3) were expressed in increased levels in DA and decreased in congenics. Interestingly, and in line with these observations, the expression levels of negative regulators of TLR signaling such as *Ptpn11* and *Pparg* was conversely increased in congenics, suggesting that the arthritis gene located within the *Cia5a* QTL might mediate the balance between activating and inhibitory signals implicated in TLR signaling.

Genes implicated in the synthesis of prostaglandins and leukotrienes (*Pla2g4a*, *Ptgs2*/*Cox2*, *Ptges*) were also increased in DA (Table 3), while genes that counteract eicosanoid-mediated inflammation (*Ptgis*, *Cyp2j3*) were increased in congenics (Table 5).

### Increased expression of anti-inflammatory genes, including nuclear receptors (NRs), in synovial tissues from DA.F344(Cia5a) Congenics

Several genes with known anti-inflammatory and cytokine-suppressing properties were expressed in increased levels in DA.F344(Cia5a) synovial tissues, and reduced in DA. These included the NRs *Lxra*, *Pparg*, *Rev-erba*, *Rora*, *Thra*, and *Thrb* (Table 5).

*Scd1* was the gene with the most significantly increased expression in DA.F344(Cia5a) congenics with a 55-fold difference compared with DA (Tables 2 and 5). *Scd1* has been shown to reduce cytokine levels and to have anti-inflammatory activity [19,20]. We have previously reported that *Scd1* is expressed in significantly reduced levels in synovial tissues from rats with severe arthritis, and increased in the synovial tissues of yet another arthritis-protected congenic strain [14].

*Adipoq* and *Timp3*, which is an inhibitor of the TNFα converting enzyme (TACE), were two additional anti-inflammatory genes expressed in significantly increased levels (>10-fold) in DA.F344(Cia5a) (Table 5).

*Scd1* and some of the other genes up-regulated in DA.F344(Cia5a) synovial tissues such as *Adipoq, Cidea, Cd36, Fabp4, Gpd1, Lpl, Lpin1, Mgst1, Plin, Pck1, Slc2a4* and *Srebf1*, are known to be inducible by NRs (Table 2 and Table 5). These observations suggest that NRs were not only expressed in increased levels but also had increased activity in synovial tissues from DA.F344(Cia5a) compared with DA.

### Genes located within the *Cia5a* interval have significantly different expression levels

75 of the 7,925 genes expressed by all samples were located within the *Cia5a* interval. 21 of these 75 had increased expression in DA synovial tissues, and 11 were increased in the congenics. 14 of these 32 differentially expressed genes had ≥2-fold-difference. *Sphk1* and *Sectm1b* were the genes contained within the *Cia5a* interval with the most significantly increased expression in DA (7.58 and 7.61-fold, respectively), while *Itgb4* and *DIgr1* and were those with the most significantly increased expression in DA.F344(Cia5a) congenics (2.77 and 2.85-fold, respectively) (Table 6). Additionally, four genes located within the *Cia5a* interval were expressed only or predominantly in DA synovium, while two other genes were expressed predominantly in DA.F344(Cia5a) (Table 6). It is conceivable that these differences in expression levels of genes located within the *Cia5a* interval could be explained at least in part by a polymorphism in the 5’ untranslated region (UTR) that affects a transcription factor binding site in cis, thus affecting transcription efficiency, or a 3’ UTR polymorphism affecting mRNA stability.

**Table 6:** Differentially expressed candidate genes located within the Cia5a interval on rat chromosome 10*

**Gene Symbol**	**Gene name**	**Entrez Gene ID**	**Difference**	**p-value§**
***Increased in DA***			***Fold DA/Cia5a***	
*Igsf7*	immunoglobulin superfamily, member 7	287813	4.42	0.00008
*Lgals3bp*	lectin, galactoside-binding, soluble, 3 binding protein	245955	3.85	0.000001
*RGD1309310*	similar to mKIAA0195 protein	303677	3.89	0.00001
*Sectm1b*	secreted and transmembrane 1B	287884	7.61	0.00001
*Slc16a3*	solute carrier family 16, member 3	80878	5.36	0.000005
*Sphk1*	sphingosine kinase 1	170897	7.58	0.0000003
	***Increased in DA.F344(Cia5a)***		***Fold Cia5a/DA***	
*Itgb4*	integrin beta 4	25724	2.85	0.0004
*RGD1311422*	similar to CG8841-PA	287822	2.57	0.002
*RGD1561778*	similar to dendritic cell-derived immunoglobulin(Ig)-like receptor 1, DIgR1	303666	2.77	0.002
*Slc25a10*	solute carrier family 25, member 10	170943	2.15	0.004
***Expressed only or predominantly in DA***			***Frequency DA:Cia5a***	
*Cd300lf*	CD300 molecule-like family, member f	287818	6:0	0.0003
*Fdxr*	ferredoxin reductase	79122	6:1	0.0047
*Cd7*	CD7 molecule	303747	6:0	0.0003
*Sectm1a*	secreted and transmembrane 1A	287885	6:1	0.0047
***Expressed only or predominantly in DA.F344(Cia5a)***				
*Aanat*	arylalkylamine N-acetyltransferase	25120	0:7	0.0047
*Hrnbp3*	hexaribonucleotide binding protein 3	287847	1:6	0.1♯

*List contains the most signficantly differentially expressed genes; § *t*-test was used to compare means for fold-difference calculations and Fisher's Exact test to compare frequencies. #not statistically significant.

### Gene targets of microRNAs (miRNA) contained within the *Cia5a* interval were not differentially expressed

**Figure 4:**
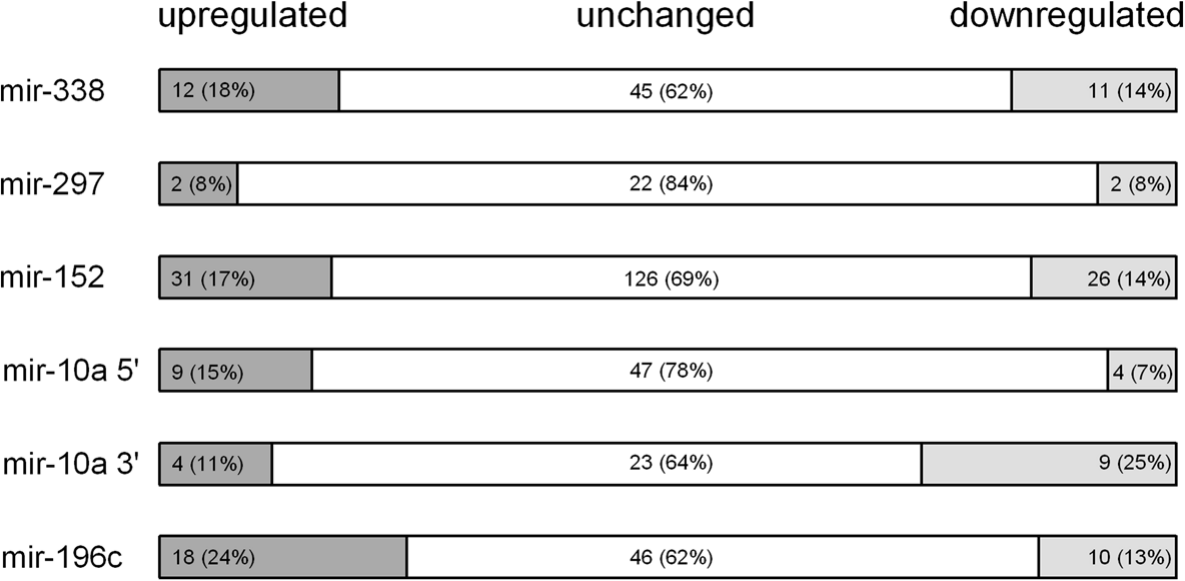
**Differentially expressed predicted targets of the miRNAs located within the *Cia5a *interval. **Six miRNAs map to the *Cia5a* interval. The number of predicted target genes of each miRNA that were up-regulated and down-regulated in DA and DA.F344(Cia5a) congenics was not statistically significant (Chi-square test with the Yates correction). Numbers (percentages intra-parenthesis) of genes expressed in increased levels (dark grey), reduced levels (light grey) or unchanged (white) in DA compared with DA.F344(Cia5a) are shown.

The *Cia5a* interval contains six predicted miRNAs. We considered the possibility that polymorphisms in one of those six miRNAs could account for the *Cia5a* effect on gene expression and arthritis severity. In that case, such polymorphism would affect the miRNA activity on the transcription of its target genes. Therefore, we look for the differential expression of targets of all six predicted miRNAs located within the *Cia5a* interval. A list of target and non-target genes was generated for each of the six miRNAs, but no significant over-representation of targets was detected (Figure 4), suggesting that polymorphisms affecting the expression or function of the miRNAs contained within the *Cia5a* interval are less likely to explain the differences in gene expression identified in this study.

### Analyses of cell type specific genes suggests synovial tissue cellularity differences between DA and DA.F344(Cia5a) Congenics

13 genes known to be specifically expressed by the cell types of interest to this study were used to compare DA and DA.F344(Cia5a) synovial tissues (Additional file 3: Table S2). The expression levels of those genes suggested increased numbers of FLS (consistent with synovial hyperplasia), macrophages, dendritic cells (DC), neutrophils and T cells in the synovial tissues of DA, compared with congenics (Additional file 3: Table S2). No gene specific for B cells, NK cells or Tregs were among those differentially expressed between the two strains, suggesting that the number of these cells in the synovial tissues of these two strains was not significantly different.

## Discussion

Disease severity and articular damage are associated with increased risk for disability, joint deformities and reduced life expectancy in patients with RA [21-23]. Yet, little is known about the genes implicated in the regulation of disease severity and articular damage genes in RA, and these genes could be the most relevant targets for new therapies aimed at preserving the joint architecture and function.

We have previously identified *Cia5a*, a 20.6 Mb arthritis severity and joint damage regulatory locus, on rat chromosome 10 [10]. *Cia5a* co-localizes with other arthritis severity loci identified in other rodent models of arthritis such as oil-induced arthritis (*Oia3*) [24], and CIA in a DAxACI intercross (*Cia27*) [25]. There have been no genome-wide association or linkage studies of disease severity and joint damage in RA, and therefore, it is unknown whether the *Cia5a* syntenic region on human chromosome 17q22-q25 harbors a severity or joint damage arthritis regulatory gene. However, the human 17q22-q25 region contains a locus previously linked with RA susceptibility [26]. In the present study we analyzed synovial tissues from DA rats, which develop severe arthritis (PIA) with pronounced synovial hyperplasia and cartilage and bone destruction, and synovial tissues from the DA.F344(Cia5a) congenics, which develop mild and non-erosive disease. These two strains share the same MHC and are genetically identical except for the presence of F344 alleles at the *Cia5a* interval, underscoring the magnitude of the effect of this single locus on clinical disease, on histologic joint damage [10] and on gene expression (present study). DA.F344(Cia5a) congenics had significantly reduced expression of genes previously implicated in RA pathogenesis, RA severity and articular damage, including *Il1b, Il18, Mif, Mmp3* and *Mmp14*. These and other similarities between DA rats and RA synovial tissues’ gene expression, such as increased expression of chemokines, matrix proteins, adhesion molecules, mediators of innate immune responses, and others, underscore and further validate the potential clinical relevance of our model and discovery strategy.

We identified a new role for *Cia5a* on the regulation of the expression of members of the *Syk* pathway, where forty-seven genes directly or indirectly related to *Syk* activation were expressed in increased levels in DA, and significantly reduced levels in DA.F344(Cia5a) congenics. *Syk* is a tyrosine kinase that phosphorylates ITAM motifs in trans-membrane receptors or adaptors, and interacts with partners like *Vav*, *PI3K* and *Slp76*[27]. *Syk* activation mediates signaling through several cell surface receptors, including those with significantly different levels in this study such C-lectin type receptors, *Fcer1g*, *Fcgr2a*, *Trem2*, *Tyrobp*, integrins, and the T-cell receptor (TCR) (Figure 3). Resident and infiltrating inflammatory cells in the RA synovial pannus, such as mast cells, macrophages, B and T cells, express these Syk-activating receptors. These resident cells and infiltrating cells have been implicated in arthritis pathogenesis and joint damage, raising the possibility that part of their effect may be mediated by Syk-activating receptors.

Analyses of cell-specific genes suggested reduced numbers of macrophages, dendritic cells, neutrophils and T cells in the synovial tissues of congenics compared with DA, which is in agreement with our previous histologic analyses and might explain part, but not all of the differences in expression of Syk genes. Additionally, DA.F344(Cia5a) congenics had significantly lower levels of the FLS-specific gene *Cdh11*, compatible with the reduced synovial hyperplasia that we have previously described.

*Syk* pathway members regulate several cellular processes implicated in arthritis pathogenesis and articular damage, ranging from the production of reactive oxygen species, NFκB activation and the transcription of pro-inflammatory mediators such as *Il1b* and *Ccl2*, to the cell proliferation required for the development of synovial hyperplasia, and actin cytoskeleton rearrangements [27]. NFκB activity is regulated by *Syk* and by several other pathways including TLRs and cytokine receptors [28]. The NFκB pathway has a central role in the production of pro-inflammatory cytokines such as IL-1β, IL-6 and TNFα, in the development of synovial hyperplasia and in disease severity [29-31]. Actin cytoskeleton rearrangements are also regulated by the Syk pathway [27], and are required for the migration of inflammatory cells into the synovial tissue, and for synovial cells and synovial tissue invasion and destruction of cartilage [13,32]. Therefore, our observations suggest that a gene located within the *Cia5a* interval is a new regulator of the expression of *Syk* pathway genes implicated in key processes in arthritis pathogenesis.

The precise mechanisms whereby *Cia5a* regulates the expression of *Syk* genes remain unclear, and might reflect differences in tissue cellularity, multiple cell-activating processes, or a polymorphism in transcription factor located within the *Cia5a* interval that affects transcription. Studies by our group of synovial tissues obtained from four different congenic strains yielded different results in gene expression (Brenner et al., manuscript in preparation) [12,14,33], suggesting that the *Syk*-regulatory effect of *Cia5a* is a specific observation, and not simply related cellularity differences or inflammation.

*Syk* has been recently implicated in arthritis pathogenesis and joint damage, and *Syk*-deficient mice are protected from autoantibody-induced erosive arthritis [34], and treatment with a SYK inhibitor significantly reduced disease severity and joint erosions and damage in collagen-induced arthritis [35]. Both the total and phosphorylated forms of SYK are expressed in increased levels in RA synovial tissues compared with osteoarthritis, and SYK inhibition reduced the expression of IL-6 and MMP-3 [17]. More importantly, the use of a SYK inhibitor significantly reduced disease activity in patients with RA [36], with 67%, 43% and 28% of patients achieving ACR20, ACR50 and ACR70, respectively, in a 3-month double-blind and placebo-controlled study [37]. Therefore, it is conceivable that the *Syk* pathway genes differentially expressed in this study could help identify patients more likely to benefit from therapy with SYK inhibitors. Additionally, *Syk* is critical to TNFα-induced responses [38], raising the possibility that the *Syk* pathway 47-gene signature could be used to predict increased TNFα activity prior to choosing a biologic therapeutic agent. Additionally, the increased expression of *Syk* pathway genes could identify patients at increased risk to develop erosive disease and could become a prognostic tool. Lastly, the *Cia5a* gene itself has the potential to become a new target for therapies aimed at reducing articular damage via inhibition of *Syk* pathway genes, including processes downstream from *Syk* such as NFκB.

While several genes with pro-inflammatory, proteolytic, innate immunity and inflamasome-related activity were expressed in reduced levels in DA.F344(Cia5a) congenics, groups of genes with known anti-inflammatory properties were expressed in increased levels in congenics. These genes included *Timp3*, *Ptpn11*, antagonists of reactive oxygen species (*Cat*, *Gss*, *Sod1*) and nuclear receptors. Nuclear receptors such as *Lxra*, *Pparg and Rora* have been shown to interfere with NFκB and AP-1 activation [39-41], and to have anti-inflammatory and arthritis-suppressive properties [42-45]. *Rxrg* was another nuclear receptor expressed in significantly increased levels in DA.F344(Cia5a) congenics. While *Rxrg* itself has not been studied in the context of arthritis, it dimerizes with *Lxra*, *Pparg*, and with *Vdr*, and is required for their anti-inflammatory activity. Additionally, several nuclear receptor-inducible genes, including the inflammation-suppressor *Scd1*[20] were expressed in increased levels in the synovial tissues of the congenics. These observations suggest that not only nuclear receptor levels were increased, but also their activity. We have recently identified a similar nuclear receptor expression signature in another arthritis-protective congenic strain, DA.ACI(Cia25) [14], suggesting that this effect is not specific to the *Cia5a* locus, but more broadly correlates with preservation of both a normal synovial environment and articular architecture.

The gene with the most significantly increased expression in DA compared with congenics was *Tnn* (Tenascin N). While little is known about this secreted extra-cellular matrix glycoprotein, it has been implicated in cancer-associated angiogenesis [46], and in integrin-dependent cancer motility [47]. Another member of the tenascin family, Tenascin C (*Tnc*), was recently shown to be an endogenous activator of TLR4, an inducer of IL-6 and TNFα, and was required for joint damage in arthritic mice [48], suggesting that *Tnn* could have a function similar to *Tnc* in arthritis.

Lastly, we considered the possibility that a polymorphism in the 5’ UTR or 3’UTR region of the gene accounting for *Cia5a* could interfere with its transcription and/or mRNA stability, respectively, leading to increased or reduced gene-specific mRNA levels. We looked for differentially expressed genes and genes preferentially expressed by only one of the strains and located within *Cia5a* as a clue to the above possibility. Thirty-eight genes met these criteria, and particularly the most significant sixteen genes are interesting candidates that will be studied in detail (Table 6).

## Conclusion

In conclusion, in the present study we identified a pattern of gene expression regulated by *Cia5a*, which included several inflammatory mediators and 47 members of the *Syk* pathway. Levels of several mediators of arthritis pathogenesis, synovial hyperplasia and articular damage were also reduced in DA.F344(Cia5a) congenics, underscoring the importance of the gene accounting for this locus. Increased expression of nuclear receptors correlated with joint preservation, and a new potential mediator of inflammation, *Tnn*, was identified for the first time in synovial tissues. Our observations suggest that the gene accounting for *Cia5a* has the potential to become an important new target for therapies aimed at preserving joint architecture free of damage, and reducing inflammation.

## Methods

### Rats

DA/BklArbNsi (DA) rats were originally purchased from Bantin & Kingman (Freemont, CA), maintained at the Arthritis and Rheumatism Branch, National Institute of Arthritis and Musculoskeletal and Skin Disease, National Institutes of Health, and then transferred to the Feinstein Institute for Medical Research (FIMR; formerly North Shore-LIJ Research Institute, Nsi). DA.F344(Cia5a) congenic rats were generated as previously described [9]. Briefly, a 20.6Mb interval from chromosome 10 from the arthritis-resistant F344 strain was introgressed into arthritis-susceptible DA rats through genotype-guided breeding (Figure 1A). This strategy selected for F344 alleles at the *Cia5a* interval while excluding donor genome contamination at other loci known to regulate arthritis [10,49]. Experiments were done with 8–12 week-old male rats homozygous at the congenic interval. All experiments were conducted under an Institutional Animal Care and Use Committee (IACUC)-approved protocol.

### PIA and tissue collection

Male DA (n=6) and DA.F344(Cia5a) (n=8) congenic rats were anesthetized and injected intradermally with 150 μl of pristane (MP Bio, Solon, OH) divided into two injection sites at the base of the tail (day 0) [50]. Arthritis severity was assessed with a previously described 80-point scoring system [51]. Ankle synovial tissues were collected 21 days post-induction of arthritis.

### RNA extraction

Total RNA was extracted from synovial tissues using the RNeasy Mini Kit (Qiagen, Valencia, CA) according to the manufacturer's instructions and including a DNase treatment step. RNA was quantified and assessed for purity using the NanoDrop spectrophotometer (Rockland, DE). RNA integrity was verified with the BioAnalyzer 2100 (Agilent, Palo Alto, CA).

### Microarray

All reagents and procedures were previously optimized for use with the Illumina Whole-Genome Expression platform [12]. Briefly, total RNA (200 ng) was amplified and biotinylated using the TotalPrep labeling kit (Ambion, Austin, TX). Each individual sample was hybridized to one individual array in the RatRef-12 Expression BeadChip (Illumina, San Diego, CA), which contains 22,522 probes covering 21,922 rat genes selected primarily from the NCBI RefSeq database (Release 16). Hybridization was done in Illumina IntelliHyb chambers, followed by washing and staining with Cy3-streptavidin. The BeadChip was scanned on a high-resolution Illumina BeadArray reader using a two-channel 0.8 μm resolution confocal laser scanner.

### cDNA synthesis and quantitative real-time PCR (qPCR) expression analysis

Differences in the expression of selected genes from the microarray analyses were validated with qPCR. The qPCR conditions have been described elsewhere [12]. Briefly, total RNA (200 ng) from each sample was used for cDNA synthesis using Superscript III (Invitrogen). Primers and qPCR probes were designed to target the same exons as the corresponding Illumina RatRef-12 Expression BeadChip probes (Additional file 4: Table S1). We used Universal ProbeLibrary (Roche, Indianapolis, IN) and Taqman (ABI, Applied Biosystems, Foster City, CA) probes labeled with FAM at the 5' end and TAMRA at 3' end. Reactions were prepared in duplicates with Eurogentec qPCR MasterMix (San Diego, CA), and run on an ABI Prism 7700 thermocycler using SDS software version 1.9.1 (ABI). *Ct* (threshold cycle) values were adjusted for *Gapdh* in each sample (Δ*Ct*). Expression levels (Δ*Ct*) were compared using the *t*-test and a p-value ≤0.01 was considered significant. Fold-differences were calculated with the 2^-ΔΔ*Ct*^ method [52].

### MicroRNAs (miRNA)

We considered the possibility that polymorphisms in a miRNA located within the *Cia5a* interval could account for the *Cia5a* effect on gene expression and arthritis severity. In that case, such polymorphism would affect the miRNA activity on the transcription of its target genes. Therefore, we looked for miRNAs mapping to the *Cia5a* interval using the miRBase [53]. Target genes for the miRNAs contained within the *Cia5a* interval were predicted with TargetScan [54]. Enrichment for differentially expressed predicted targets of miRNAs located within *Cia5a* was calculated using the Chi-square test with the Yates correction.

### Cellular subset gene expression signatures

Differences in tissue resident and infiltrating cell populations can affect the interpretation of gene expression analyses. We looked for cell-specific genes using the GNF Mouse GeneAtlas V3 (Affymetrix MOE430, GEO code GSE10246), a database containing gene expression information for 96 resting and stimulated mouse cell types and tissues [55,56], as well as the BioGPS website (http://www.biogps.org, Scripps Research Institute). The GNF Mouse GeneAtlas V3 did not include fibroblast-like synoviocytes (FLS) or regulatory T cells (Tregs). Therefore, additional non-redundant cell signature genes were obtained from the literature to represent FLS and Tregs [57-65]. We generated a list of genes specific for B cells, T cells, Treg cells, NK cells, FLS, dendritic cells, mast cells, macrophages and neutrophils. We next looked for those cell-specific genes within the list of genes differentially expressed between DA and DA.F344(Cia5a) congenics, as well as in the list of genes preferentially expressed, or only expressed in one strain and not in the other in order to gain insight into differences in cell populations.

### Microarray analysis and statistics

Microarray fluorescence intensities were extracted using BeadStudio 2.0 (Illumina). Fluorescent intensities were background-subtracted and then normalized using the cubic spline algorithm. Normalized data were log_2_-transformed prior to all analyses. Probes consistently expressed in all arrays were included in the analyses. Genes with ≥1.5-fold difference in intensity between DA and DA.F344(Cia5a) and a *t*-test p-value ≤ 0.01 were considered differentially expressed and selected for pathway detection analyses using IPA 5.5.1 (Ingenuity Systems, Redwood City, CA), as well as public online databases (Ensembl, Genecards, Oncomine, BioGPS, Rat Genome Database) and literature search (Pubmed).

Strain-specific (genes only in one of the strains), or preferential strain (genes expressed in a higher percentage of rats of one strain, and in lower percentage of rats of the other strain) gene expression was determined with the Fisher’s exact test.

Enrichment for biological functions and disease groups was determined with the IPA software and calculated using the Fisher’s exact test with the Benjamini-Hochberg correction and a cutoff p-value of ≤0.05. Enrichment for differentially expressed genes within specific cell subsets, or genes located within the *Cia5a* interval was calculated using the Fisher’s exact test. Non-normally distributed arthritis severity scores were compared using the Mann–Whitney test.

Funded by a Postdoctoral Fellowship Award from the New Jersey Chapter of the Arthritis Foundation to Dr. M. Brenner, and by the National Institutes of Health grants R01-AR46213, R01-AR052439 (NIAMS) and R01-AI54348 (NIAID) to Dr. P. Gulko.

## Competing interests

The authors have no competing financial interests to declare. The results presented in this manuscript are the basis for a recently submitted patent application.

## Authors’ contributions

MB carried out the work with rats, including induction of arthritis, tissue dissection and all the steps in the microarray experiments, including a significant role in the analyses, interpretation of the results and manuscript writing. PSG conceived and designed the study and did the statistical and pathway analyses analysis, as well as the manuscript writing. Both authors read and approved the final manuscript.

## Supplementary Material

Additional file 1 **Table S3. **Functional categories related to angiogenesis and extra-cellular matrix turnover that were significantly down-regulated in DA.F344(Cia5a) synovium. Click here for file

Additional file 2 **Table S4. **Functional categories related to pro-inflammatory signals, chemotaxis, and activation of myeloid cells that were significantly down-regulated in DA.F344(Cia5a) synovium*. Click here for file

Additional file 3 **Table S2. **Detection frequency and expression values of cell subset specific genes in DA and DA.F344(Cia5a) synovial tissues. Click here for file

Additional file 4 **Table S1. **Primers and probes used for qPCR and the exons they targeted. Click here for file
