# Supplementary material for: The arthritis severity locus Cia5a regulates the expression of inflammatory mediators including Syk pathway genes and proteases in pristane-induced arthritis
Source: BMC Genomics. 2012 Dec 19;13:710. doi: 10.1186/1471-2164-13-710 (PMC3548698; doi:10.1186/1471-2164-13-710)
Supplement: Additional file 1 — Table S3. Functional categories related to angiogenesis and extra-cellular matrix turnover that were significantly down-regulated in DA.F344(Cia5a) synovium. [file 1471-2164-13-710-S1.pdf]

Supplemental table 3. Functional categories related to angiogenesis and extra-cellular matrix turnover that were significantly down-regulated in DA.F344(Cia5a) synovium.

Function	Genes	*p*-value [a]	Selected genes
Development of blood vessel	67	8.98×10^{-6}	*Chm, Col1a1, Col3a1, Col5a1, Fes, Foxm1, Furin, Lox*
angiogenesis	49	1.75×10^{-3}	*Adamts1, Itgav, Itgb2, Nos3, Plat, Tgfb1, Tie1, Timp1, Tnfrsf12a*
ECM deposition and fibrosis [b]			
organization of filaments	22	6.34×10^{-3}	*Anxa2, Col1a1, Col3a1, Col5a1, Col5a2, Col11a1, Fat1, Prc1*
synthesis of glycosaminoglycan	10	4.75×10^{-3}	*B3gat3, B4galnt1, B4galt7, Has1, Pdgfrb, Tgfb1, Ugdh*
quantity of glycosaminoglycan	6	1.94×10^{-3}	*Chsy3, Gusb, Has2, Ndst2, Plau*
fibrosis	37	1.87×10^{-3}	*Ifngr1, Ifngr2, Mdk, Pdgfrb, Pdpn, Tgfb1, Timp1*
closure of wound	7	9.16×10^{-3}	*Fap, Itga5, Itgav, Itgb2, Mmp9, Serpine1, Tgfb1*
Destruction of cartilage tissue	6	7.32×10^{-3}	*Fcgr1a, Fcgr2a, Ikbkb, Il18, Il1b, Slpi*
cleavage of gelatin	6	1.01×10^{-2}	*Ctsk, Ctss, Mmp3, Mmp9, Mmp14, Mmp19*
Remodeling of tissue	22	6.00×10^{-4}	*Hmox1, Mmp9, Mmp14, Pdgfrb*
remodeling of bone	18	1.65×10^{-3}	*Enpp1, Sparc*
resorption of bone	16	5.85×10^{-3}	*Ca2, Cd44, Ctsk, Fcer1g, Lgmn, Ptafr, Ptk2b, Tpp1, Tyrobp*

[a] Fisher's exact test with Benjamini-Hochberg correction

[b] ECM: extracellular matrix
