# Supplementary material for: The arthritis severity locus Cia5a regulates the expression of inflammatory mediators including Syk pathway genes and proteases in pristane-induced arthritis
Source: BMC Genomics. 2012 Dec 19;13:710. doi: 10.1186/1471-2164-13-710 (PMC3548698; doi:10.1186/1471-2164-13-710)
Supplement: Additional file 2 — Table S4. Functional categories related to pro-inflammatory signals, chemotaxis, and activation of myeloid cells that were significantly down-regulated in DA.F344(Cia5a) synovium*. [file 1471-2164-13-710-S2.pdf]

Supplemental Table 4. Functional categories related to pro-inflammatory signals, chemotaxis, and activation of myeloid cells that were significantly down-regulated in DA.F344(Cia5a) synovium *.

Function	Genes	*p*-value [a]	Selected genes
Inflammatory mediators			
IκB kinase/NF-κB cascade	19	3.81×10^{-3}	*Ikbkb, Myd88, Nfkb1, Nfkb2, Pdcd11, Tifa, Tlr2, Tlr6*
degranulation of mast cells	16	1.67×10^{-4}	*Fcer1g, Fyn, Lyn, Plcg2, Syk*
generation of PGE2 [b]	9	9.18×10^{-3}	*Pa2g2d, Pla2g4a, Tbxas1, Ptgs2/Cox2, Ptges*
acute phase reaction	8	1.87×10^{-3}	*Il1b, Il6r, Lbp, Orm2*
Recruitment and accumulation			
cell movement	137	4.47×10^{-10}	*Bcr, Cdh11, Ezr/Vil2, Furin,Thy1*
adhesion of cells	107	6.28×10^{-6}	*Bcr, Bgn, Sdc1, Thbs2, Vcan*
migration of leukocytes	63	2.40×10^{-5}	*Cd44, Clec11a, Clec4m, S100A8*
chemotaxis	61	3.54×10^{-4}	*Ccl6, Ccl7, Ccr2, Ccr5, Ccr6, Cxcl2, Cxcl13, Cxcr4*
adhesion of leukocytes	38	2.37×10^{-4}	*Itga5, Itgam, Itgav, Itgb2, Itgb7*
recruitment of phagocytes	22	2.34×10^{-4}	*Cd44, Cd8a, Il18, Il1b, Il6r, Thbs2*
infiltration of neutrophils	22	2.68×10^{-4}	*Adrbk1, Pf4, Ptgs2*
accumulation of phagocytes	18	3.48×10^{-4}	*Grn, Mmp3, Mmp9*
cell rolling of leukocytes	13	2.47×10^{-3}	*Clec7a, Pf4, Sele, Selp*
activation of endothelial cells	8	4.66×10^{-3}	*C3, Il1b, Il6r, Mmp9, Plau*
transmigration of monocytes	7	9.16×10^{-3}	*Col1a1, Col4a1, Ctss, Mmp9, Tgfb1*
Activity			
phagocytosis of cells	37	1.82×10^{-9}	*C3, C1qa, Clec7a, Colec12, Fcer1g, Fcgr1a, Fcgr2a*
activation of APC [c]	24	5.60×10^{-3}	*Fcgr1a, Fcgr2a, Fcgr3a, Lbp, Ltb,Tlr2, Trem2*
respiratory burst	19	8.12×10^{-6}	*Cyba, Ncf1, Ncf2, Ncf4*
apoptosis of phagocytes	19	3.48×10^{-3}	*Birc3, C1qa, Cd14, Dffa, Hif1a, Il18, Mif, Pla2g4a*
apoptosis of granulocytes	15	2.13×10^{-3}	*Bak1, Bcl2a1, Casp3, Il1b, Inpp5d, Itgam, Itgb2*
processing of antigen	12	5.81×10^{-7}	*Cd74, Ctse, Ifi30, RT1-DMa, -DMb, -DQa1, -DRa, -DRb1*

* There was significant overlap among genes of each functional group, and some genes are listed in more than one category.

[a] Fisher's exact test with Benjamini-Hochberg correction

[b] PGE2: prostaglandin E2

[c] APC: antigen-presenting cells
