# Supplementary material for: The arthritis severity locus Cia5a regulates the expression of inflammatory mediators including Syk pathway genes and proteases in pristane-induced arthritis
Source: BMC Genomics. 2012 Dec 19;13:710. doi: 10.1186/1471-2164-13-710 (PMC3548698; doi:10.1186/1471-2164-13-710)
Supplement: Additional file 3 — Table S2. Detection frequency and expression values of cell subset specific genes in DA and DA.F344(Cia5a) synovial tissues. [file 1471-2164-13-710-S3.pdf]

Supplemental Table 2. Detection frequency and expression values of cell subset specific genes in DA and DA.F344(Cia5a) synovial tissues[¶].

**Cell type**	**Entrez symbol**	**Detected in DA (n=6)**	**Detected in Cia5a (n=8)**	**DA avg signal**	**Cia5a avg signal**	**Fold DA/Cia5a[§]**	***p*-value (t-test)[§]**	**Expression***
**synovial fibroblast**	Cdh11	6	6	243	136	1.87	0.003	DA
**B cell**	*None different*	-	-	-	-	-	-	-
**T cells**	Cd3g	6	8	798	137	-6.91	0.00001	DA
	Cd6	6	5	130	49	-2.59	0.0003	DA
	Cd3d	6	2	412	170			DA
	Cd3e	6	1	181	77			DA
**dendritic cell**	Cd209d	6	8	1539	246	-9.24	0.0001	DA
**dendritic cell, plasmacytoid**	Siglech	6	3	131	112			DA
**macrophage**	Cd14	6	8	8126	3888	-2.13	0.001	DA
**neutrophil**	Fcnb	6	8	3525	782	-9.74	0.001	DA
**mast cell**	Fcer1a	3	8	94	255			Cia5a
	Ahrr	1	5	51	59			Cia5a
	1810011H11Rik	6	1	153	107			DA
	Mcpt-ps1	6	8	288	717	2.43	0.001	Cia5a

¶ = some these genes are expressed in tissues unrelated to the synovial tissue or myeloid and lymphoid cells.
# "Detected in" = numer of dynovial samples that expressed the specific gene.
* Expression=strain with increased or predominant expression of the specific gene.
