# Supplementary material for: The arthritis severity locus Cia5a regulates the expression of inflammatory mediators including Syk pathway genes and proteases in pristane-induced arthritis
Source: BMC Genomics. 2012 Dec 19;13:710. doi: 10.1186/1471-2164-13-710 (PMC3548698; doi:10.1186/1471-2164-13-710)
Supplement: Additional file 4 — Table S1. Primers and probes used for qPCR and the exons they targeted. [file 1471-2164-13-710-S4.pdf]

Supplemental Table 1. Primers and probes used for qPCR and the exons they targeted.

Gene	Forward primer	Reverse primer	Probe [1]	Target Exon			
				RatRef-12 [2]	FP	Probe	RP
Tnn	AGCTGTCGGTTGGGAAATAC	TGTGAACTTCCAACCATTGTG	98	22 (3' UTR)	20	20	21
Mmp3	GAGAACTTTCCAGGCATTGG	CCGCTGAAGAAGTAAAGAAACC	89	10 (3' UTR)	9	9	10
Il1b	CAGCAGCATCTCGACAAGAGC	AAGACATAGGTAGCTGCCACAGC	TCAGGAAGGCAGTGTCACTCATTGTGG	7 (3' UTR)	4	4	4
GAPDH	GAACGGGAAGCTCACTGGC	GCATGTCAGATCCACAACGG	TGGCCTTCCGTGTTCCTACCCCC	various	7	7	7
Pparg	TTTATAGCTGTCATTATTCTCAGTGGA	CGGGTGGTTCAGCTTCAG	81	7	6	7	7
Rxrg	CAAGCAGAAGTATCCAGAACAGC	ATCCAATGGAGCGCAGAG	84	10	9	10	10
Scd1	GAAGCGAGCAACCGACAG	GGTGGTCGTGTAGGAACTGG	125	6	1	1	2
Syk	GATGTCTGGAGCTTCGGAGT	CAGTCACTTCGCTCCCTTTC	29	15 (3' UTR)	13	13	14
Vav1	GGGTGACATCATCAAGATCCTC	GTTAGAGGGAAACCAGCCAAT	56	27 (3' UTR)	26	26	27
Tyrobp	TGGCTGAGACTGAGTCACCTT	TGCCTCTGTGTGTTGAGGTC	128	5	4	4-5	5

[1] Roche Universal Probe Library number

[2] RatRef-12 BeadChip probe.
